# Prediction of grain structure after thermomechanical processing of U-10Mo alloy using sensitivity analysis and machine learning surrogate model

**DOI:** 10.1038/s41598-022-14731-8

**Published:** 2022-06-28

**Authors:** Yucheng Fu, William E. Frazier, Kyoo Sil Choi, Lei Li, Zhijie Xu, Vineet V. Joshi, Ayoub Soulami

**Affiliations:** Pacific Northwest National Laboratory, P.O. Box 999, Richland, WA 99352 USA

**Keywords:** Energy science and technology, Nuclear fuel, Materials science

## Abstract

Hot rolling and annealing are critical intermediate steps for controlling microstructures and thickness variations when fabricating uranium alloyed with 10% molybdenum (U-10Mo), which is highly relevant to worldwide nuclear non-proliferation efforts. This work proposes a machine-learning surrogate model combined with sensitivity analysis to identify and predict U-10Mo microstructure development during thermomechanical processing. Over 200 simulations were collected using physics-based microstructure models covering a wide range of thermomechanical processing routes and initial alloy grain features. Based on the sensitivity analysis, we determined that an increase in rolling reduction percentage at each processing pass has the strongest effect in reducing the grain size. Multi-pass rolling and annealing can significantly improve recrystallization regardless of the reduction percentage. With a volume fraction below 2%, uranium carbide particles were found to have marginal effects on the average grain size and distribution. The proposed stratified stacking ensemble surrogate predicts the U-10Mo grain size with a mean square error four times smaller than a standard single deep neural network. At the same time, with a significant speedup (1000×) compared to the physics-based model, the machine learning surrogate shows good potential for U-10Mo fabrication process optimization.

## Introduction

Thermomechanical processing is critical for controlling alloy grain structures to achieve the desirable performance^1,2^. Depending on the alloy types and optimization goals, the processes used can vary drastically^3–5^, and different physics-based computational models have been developed to simulate those processes. The vertex^6,7^, phase-field^8,9^, level set^10^, and Potts models^11–13^ can be used to predict alloy grain recrystallization and growth during annealing. On the other hand, the finite element method (FEM) is suitable for analyzing stress and strain induced by hot rolling within polycrystalline microstructures^14–16^. As a promising low-enriched uranium fuel used in US high power research reactors, the monolithic fuel, uranium alloyed with 10% molybdenum (U-10Mo) contains 20% less ^235^U than conventional highly enriched uranium^17,18^; therefore, it poses less risk from a nuclear proliferation perspective^19^. It also has higher energy density and more desirable irradiation properties than other low enriched candidates for these reactors^20,21^. In monolithic U-10Mo fuel foil fabrication, multi-pass hot rolling and annealing are critical thermomechanical steps used to reduce foil thickness, release residual stress and prevent clad thinning by promoting a fine, equiaxed microstructure^1,22,23^. The FEM method can capture the complex deformation or fracture behavior that occurs during the U-10Mo rolling process. The influence of secondary phases such as uranium carbide (UC) on the rolling results can also be incorporated into the FEM model^24^. For the annealing process, modeling grain recrystallization and growth is of particular interest because grain structure affects mechanical behavior; examples include the Hall–Petch effect^25^ and the potential that variations in grain structure within the foil may cause the so-called “orange peel” effect^26,27^. Among available methods, the kinetic Monte Carlo (KMC) Potts model works well for large-scale simulations. Compared to methods such as phase-field modeling, the Potts model assumes sharp interfaces between individual grains, which simplifies the process of segmenting microstructures when analyzing simulation results^13^. These features make the Potts model suitable for communicating data with the FEM method.

The physics-based models explicitly resolve grain topologies to track their evolution over time. By resolving the micro-scale material structures, the coupled FEM-Potts model can predict U-10Mo grain structure evolution after multiple successive passes of hot rolling and annealing. A disadvantage of this technique is that the high resolution provided by the model significantly increases the simulation time and therefore its cost. The simulations can run from hours to days depending on the simulated domain size and processing (hot rolling and annealing) conditions. The high-dimensional process parameter space and the complexity of the involved physics make it difficult to identify the key factors for thermomechanical processing optimization. One way to mitigate this issue is to integrate a surrogate model for grain prediction to reduce the computational cost and efficiently identify influential factors. Efforts had been reported in which a machine learning surrogate model is used to predict alloy grain structures; this approach generally involves only a single processing step^28–31^. However, the prediction of grain structure for U-10Mo remains challenging because of the coupling of the multi-pass hot rolling and annealing processes. The presence of UC particles within the U-10Mo fuel foil also results in particle stimulated nucleation of recrystallized grains^24^. The degree to which such behavior can ultimately change the microstructure has not been well quantified.

**Figure 1:**
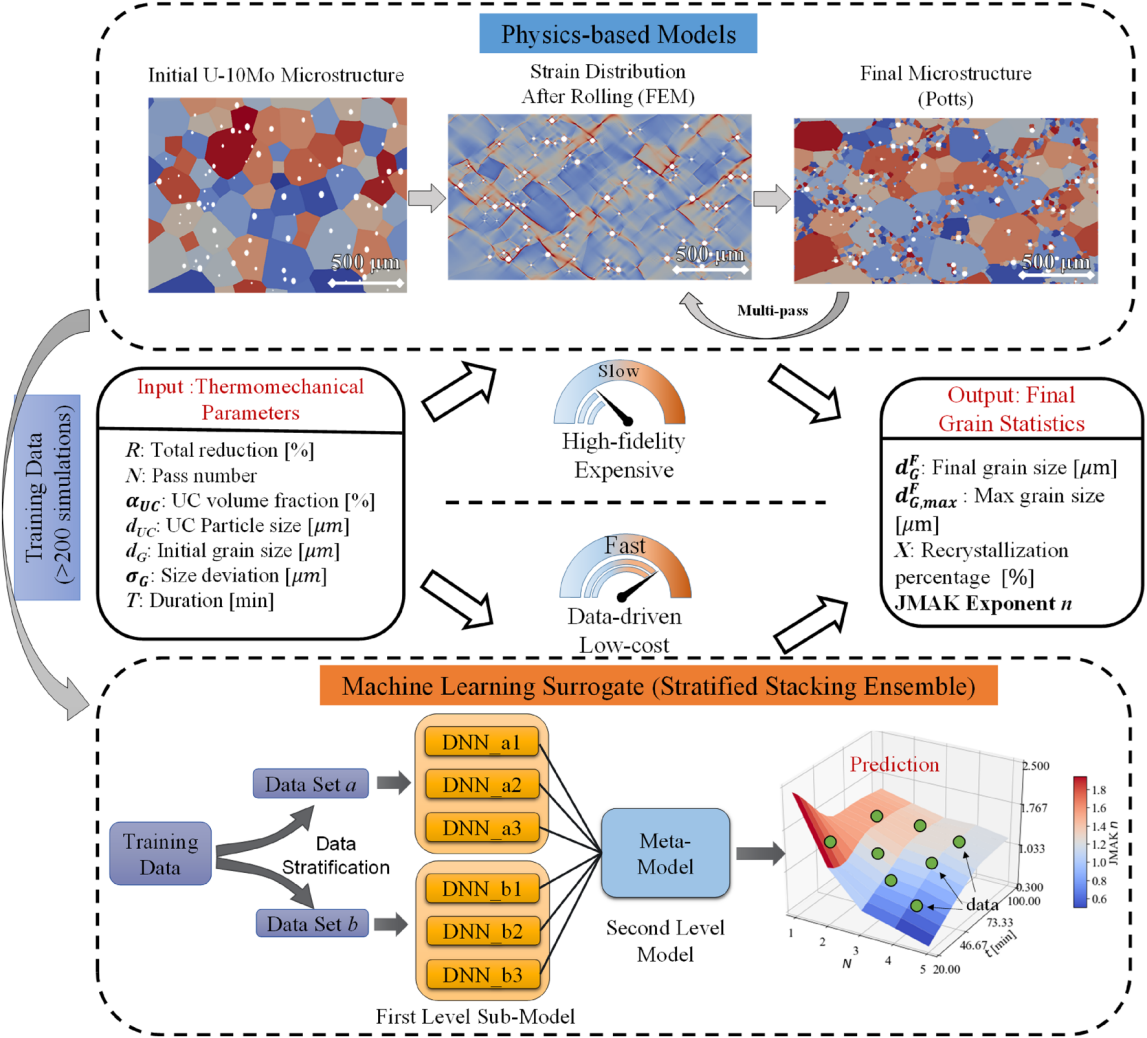
Illustration of the proposed strategy for combining physics-based simulations and a data-driven surrogate model for sensitivity analysis and fast U-10Mo microstructure prediction and optimization.

The data-driven surrogate model can aggregate high-fidelity results from numerical models and experiments, requiring no prior knowledge of the physics involved^32,33^. This is suitable for complex and nonlinear systems, which cannot be modeled faithfully with first-principles or empirical correlations. With proper training, data-driven approaches have proven their high-throughput capacity, effectiveness, and accuracy in various applications, such as fluid dynamics^34,35^, materials^36–38^, environment^39,40^, and energy^41,42^. Among existing methods, the stacking ensemble surrogate is advantageous for complex systems with low model bias and high flexibility in sub-level model selections^43^. In this work, we proposed a stratified stacking ensemble surrogate model to predict U-10Mo grain structures. Compared to traditional deep neural networks (DNN) or standard stack ensemble methods, the proposed model can select the set of submodels depending on the final grain size features and thus produce more accurate predictions for the drastic grain size change and fast recrystallization kinetics that occur during thermomechanical processing. The details are summarized in Fig. 1. For simulation designs, seven input parameters that describe the initial U-10Mo microstructures and key hot rolling and annealing settings are identified. Those parameters will be fed into the FEM-Potts models for multi-pass hot rolling and annealing. The generated final U-10Mo microstructures will provide the output of the final average grain size ${d}_{G}^{F}$, maximum grain sizes ${d}_{G,max}^{F}$, recrystallization percentage *X*, and Johnson–Mehl–Avrami-Kolmogorov (JMAK, also known as the Avrami equation)^44^ exponent *n* for representing the grain structures. Over 200 simulation datasets had been generated from the physics-based models for training and developing data-driven, machine-learning surrogate models.

The multivariate adaptive regression spline (MARS) model was trained to identify the influential factors in U-10Mo foil processing. The trained MARS model calculates the sensitivity score for each input parameter to quantitatively assess the importance of each input parameter for the final U-10Mo microstructures. Combined with the sensitivity analysis, the proposed data-driven surrogates can rapidly predict the U-10Mo final grain properties and optimize the process for unseen input conditions by selecting the most relevant input parameters.

## Results and discussion

### Single-factor analysis

**Figure 2:**
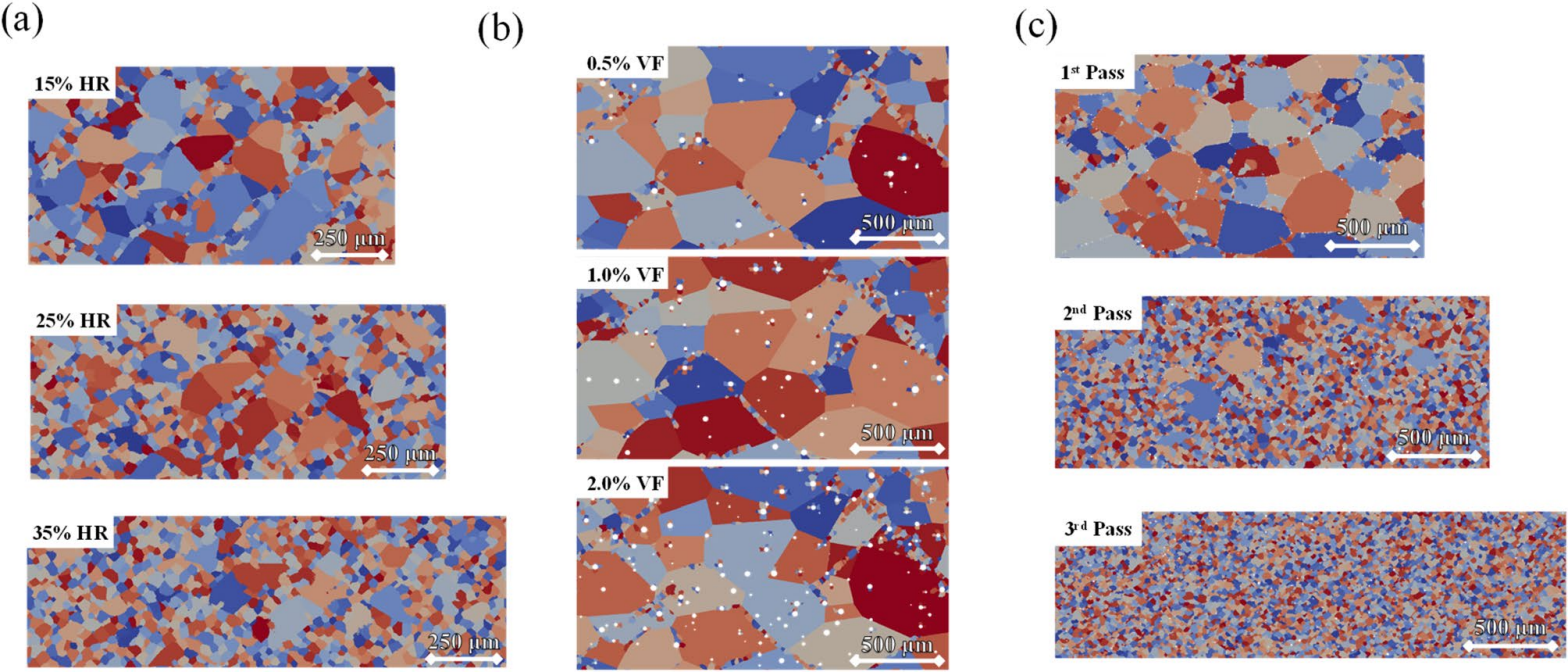
Visualization of FEM-Potts model simulated final microstructures. (**a**) One-pass hot rolling with reduction percentages of 15%, 25%, and 35%. (**b**) One-pass hot rolling with UC particle volume fractions at 0.5%, 1.0%, and 2.0% and a fixed reduction percentage of 15%. (**c**) Multi-pass hot rolling and annealing. The reduction percentage was 15% for each pass, with a 39% reduction after three passes. In these images, white regions represent UC particles. HR is hot rolling; VF is volume fraction. The color is assigned randomly to differentiate the grains.

Examples of U-10Mo microstructures from FEM-Potts simulations are shown in Fig. 2. We examined the effects of rolling reduction, UC particles, and processing passes in three subsets of images as a single-factor analysis. All the presented cases were annealed for 20 min after each rolling pass. The 15%, 25%, and 35% hot rolling reduction effect is examined in Fig. 2a, using an averaged initial microstructure size of 100 μm. The recrystallized area fraction increases with increased reduction percentage, and more refined grains appear in the microstructures. Large grains can remain even at a 35% reduction for the single-pass case. Figure 2b shows the effects of UC particles with volume fractions of 0.5%, 1.0%, and 2.0% using an initial grain size of 400 μm. The image shows that particle stimulated nucleation sites can increase as UC particle sizes increase. However, the overall area percentage of recrystallization induced by particles remains small at an annealing time of 20 min. Figure 2c shows an example of the microstructure evolution with three consecutive passes of hot rolling and annealing. Each pass has a 15% rolling reduction followed by annealing for 20 min at 700 °C. After the first pass, a relatively small area fraction has been replaced by recrystallized grains, mostly distributed along the grain boundaries. The second pass further refines the grains, leaving only a few coarse grains. After the third pass, nearly all the area has recrystallized to fine, equiaxed grains with a final average size of around 30 μm.

The single-factor analysis can infer that the reduction and processing passes are the two most influential factors in determining grain structures. At the same time, the UC particles have a very small overall effect on the final grain size and recrystallization kinetics. It should be noted that those examples only qualitatively describe the relationship between the input parameters and the final microstructure in the local design space. A more throughout understanding should be established using the global sensitivity analysis described below, which will provide a more systematic analysis of the investigated input parameter space.

### Global sensitivity analysis

**Figure 3:**
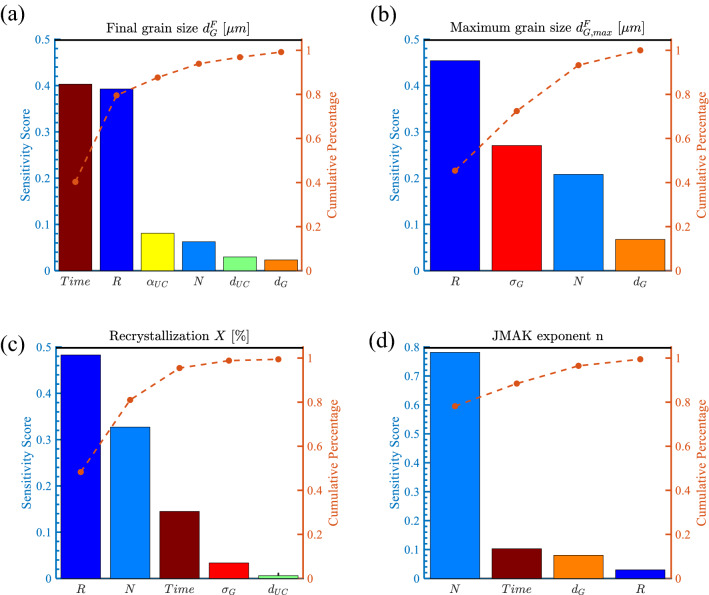
Pareto plots showing the sensitivity score ranking of the simulation parameters on the final U-10Mo alloy microstructure. (**a**) Final grain size ${d}_{G}^{F}$, (**b**) maximum grain size ${d}_{G,max}^{F}$, (**c**) recrystallization percentage $X$, and (d) JMAK exponent *n*.

For the complex U-10Mo fabrication process, the global sensitivity analysis is necessary to quantitatively rank the importance of each input parameter to guide the U-10Mo processing procedures design and optimization. By identifying crucial input variables and making non-sensitive parameters constant, the cost of modeling U-10Mo hot rolling and annealing process can be reduced significantly. The sensitivity score ${s}_{i}$, as defined in Eq. (7), is calculated for each input variable for accessing the global influence of the processing conditions and initial microstructures. Pareto plots ranking the parameters that most strongly affect the final microstructure statistics are shown in Fig. 3. In the charts, the vertical, left-side axes show scaled sensitivity scores, with the sum of ${s}_{i}$ equal to 1. The bars in the plot represent the normalized sensitivity scores for the input parameters, ranked in descending order from left to right. The right-side, vertical axes of each chart shows the cumulative percentage of the sensitivity scores. The input parameters selected in the plots contribute 99% of the output parameter variations.

For the final grain size ${d}_{G}^{F}$, the annealing time and rolling reduction percentage are the most dominant factors in determining the final grain size. Recrystallization can happen in local regions induced by concentrated strain for short annealing times. Over a long annealing time, grain coarsening becomes more dominant as grain recrystallization increases. Those effects make the annealing time an important factor in controlling the final average U-10Mo grain size. The hot rolling reduction percentage is another critical factor that determines the final average grain size for either single- or multi-pass hot rolling and annealing. For the maximum grain size, the reduction percentage, number of passes, initial grain size, and standard deviation are the four most influential factors, accounting for over 99% of the variation. In the current simulations, the maximum annealing time is 120 min. With this range, the grain coarsening effect is not dominant, thus, the contribution of annealing time to the maximum grain size is trivial. Further increasing annealing time, the abnormal grain growth will start and it would become the important mechanism that determines the final maximum grain size.

Regarding recrystallization kinetics, the dominant input parameters that affect the recrystallization and the JMAK exponent are quite different. For the recrystallization percentage, hot rolling reductions create dislocations that drive the grain refinement process. Annealing subsequently releases those dislocation sources, which can negatively or positively contribute to the final grain size, depending on the duration of the heat treatment. Along with the number of processing passes, hot rolling and annealing largely determine the final extent of U-10Mo grain recrystallization. However, for the JMAK exponent *n*, the variation can be explained by the number of passes. According to the global sensitivity analysis, the effect of UC particles on the final average grain size and recrystallization kinetics is marginal. This result is consistent with the FEM-Potts modeled microstructures shown in Fig. 1.

### Grain structure predictions

**Figure 4:**
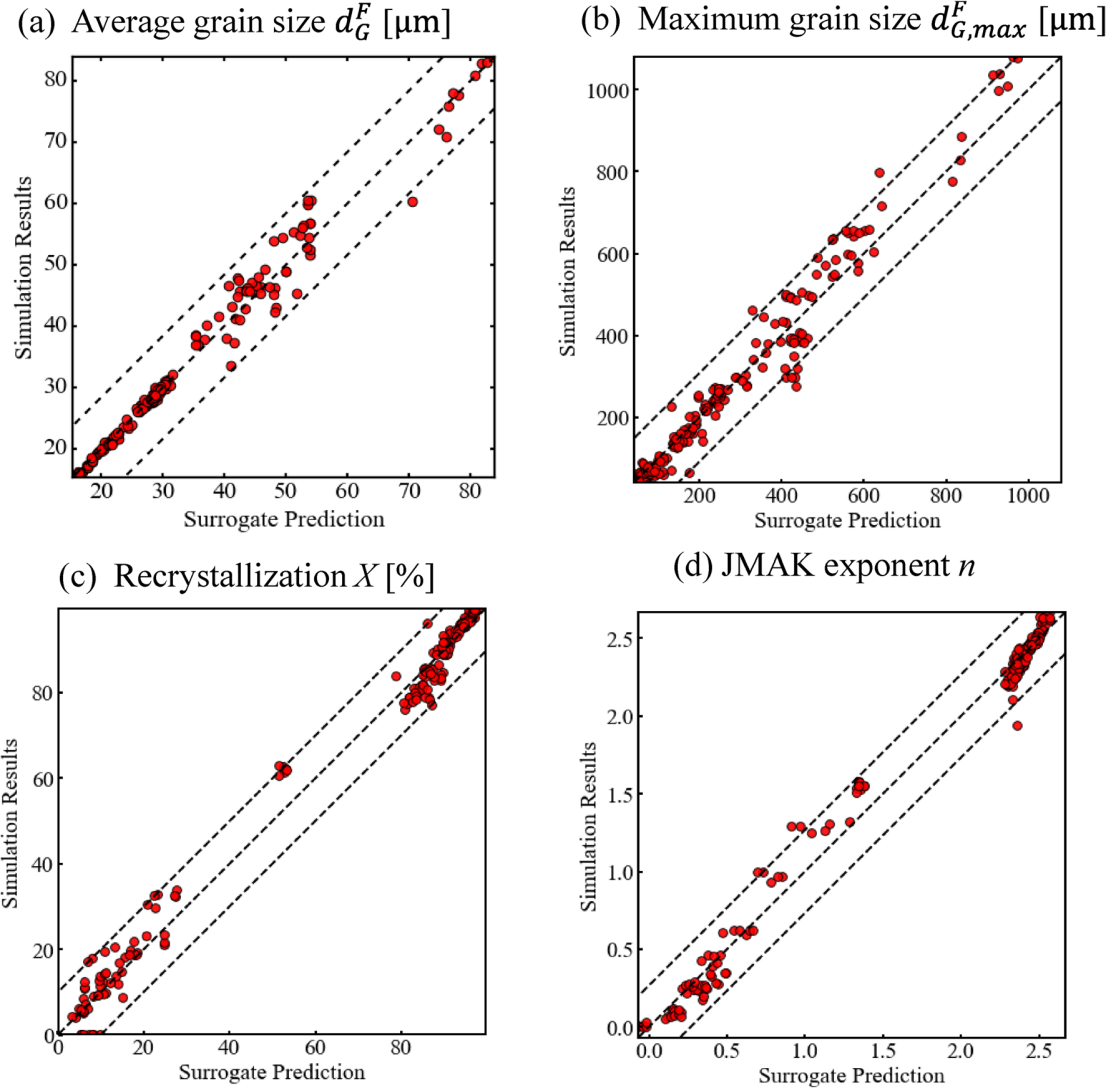
Comparison of stratified stacking ensemble model versus the FEM-Potts model simulations for the processed U-10Mo alloy (**a**) final averaged grain size ${d}_{G}^{F}$, (**b**) maximum grain size ${d}_{G,max}^{F}$, (**c**) recrystallization *X* and (**d**) JMAK exponent *n*.

By adopting machine-learning surrogates to predict U-10Mo grain structures, the high cost of the physics-based models can be mitigated while achieving real-time predictions. The performance of three different data-driven surrogates is compared in Table 1. The mean absolute error (MAE) between the surrogate model prediction and the physics-based model is calculated for each output parameter. The DNN model has the same structures for building first-level submodels in the stacking ensemble method. Table 1 shows that a single DNN has the largest MAE for all four output parameters. With the stacking ensemble method, the prediction accuracy for ${d}_{G}^{F}$ and *X* are significantly improved, while the ${d}_{G,max}^{F}$ and JMAK exponent *n* show relatively small improvement. The MAE has been further reduced with the proposed stratified stacking ensemble surrogate for all the four output parameters. To further validate the stratified stacking ensemble method, the prediction results have been compared with the FEM-Potts model simulations, as shown in the Fig. 4 parity plots.

**Table 1:** Comparison of MAE among the three tested surrogate models.

MAE	DNN	Stacking ensemble	Stratified stacking ensemble
Avg Grain Size ${d}_{G}^{F}$ [μm]	5.4	1.9	1.3
Max Grain Size ${d}_{G,max}^{F}$ [μm]	121.94	84.93	41.71
Recrystallization X [%]	12.10	3.40	2.55
JMAK exponent *n*	0.1	0.087	0.056

The average and maximum U-10Mo grain sizes are scattered across their parameter space. The recrystallization percentage *X* and JMAK exponent *n* are more polarized because of the drastic change in recrystallization kinetics and multi-pass processing. The stratified stacking ensemble surrogate achieves good prediction accuracy across the parameter space for all four output parameters fields. The relative errors are below 20%, as indicated by the dashed boundary lines.

**Figure 5:**
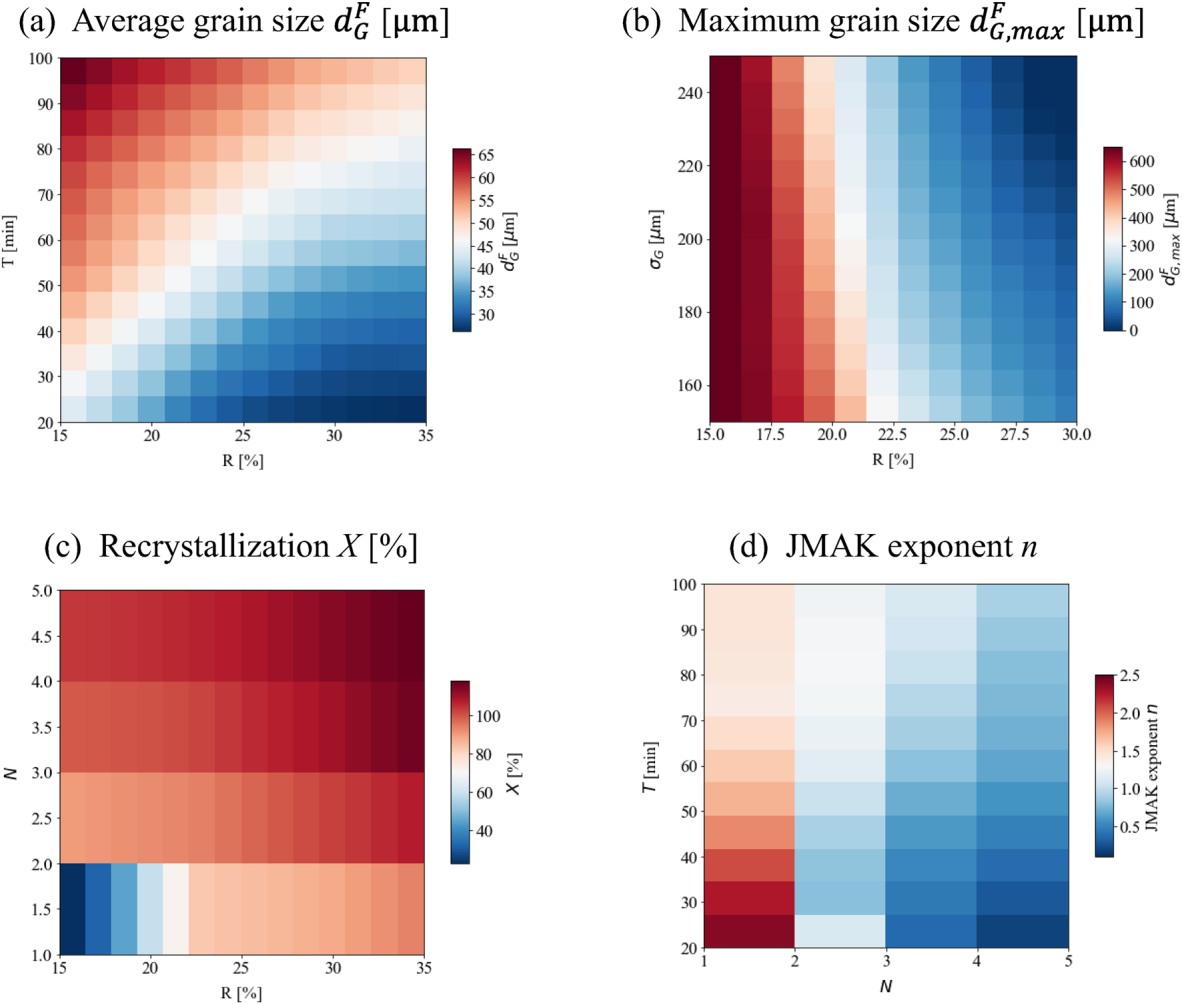
Contour plots for the four predicted output parameters: (**a**) ${d}_{G}^{F}$, (**b**) ${d}_{G,max}^{F}$, (**c**) *X*, (**d**) JMAK exponent *n*, to their corresponding two most sensitive input parameters identified by the sensitivity analysis.

The most influential parameters in predicting the final microstructure can be identified by combining the surrogate model with the sensitivity analysis. Example contour plots are shown in Fig. 5 for those most important inputs.

As shown in Fig. 5a, the final grain size is strongly correlated with *R* and *T* in the investigated range. The response has a steeper slope along the annealing time *T* axis than along the reduction percentage *R* axis. Therefore, the annealing time *T* exhibits the most dominant effect in determining the final grain size ${d}_{G}^{F}$, which is suggested by the sensitivity analysis in Fig. 3a. For the maximum grain size ${d}_{G,max}^{F}$ (Fig. 5b), reduction percentage *R* and standard deviation *σ*_*G*_ are the top two influential factors as indicated by Fig. 3b. For the selected base conditions, reduction percentage has the strongest effect on ${d}_{G,max}^{F}$. Increasing the reduction percentage from 15 to 35% significantly suppresses abnormal grain growth. For recrystallization (Fig. 5c), the lowest recrystallization percentages (15%) occur in the single-pass case. The recrystallization percentage *X* would be close to 100% for either multi-pass or large reductions. Finally, the JMAK exponent *n* versus *N* and *T* is plotted in Fig. 5d. The dependence of the JMAK exponent *n* on the two most critical parameters *N* and *T* is more complicated than the final grain size. The presence of UC particles and the distribution of initial microstructures both can affect JMAK correlation predictions. For single-pass processing, *n* is in the 1.4–2.5 range. With an increase of processing passes, *n* decreases accordingly. In general, the JMAK equation describes one continuous process of annealing. In multi-pass cases, *n* should be treated as an effective coefficient to account for the multi-pass effect. This results in a much smaller *n* compared to single-pass cases.

In summary, we developed a machine-learning surrogate model to explore the hot rolling and reduction influence on U-10Mo grain microstructures. The sensitivity analysis identified the most critical factors that affect U-10Mo final microstructures, and the surrogate model can provide grain structure predictions 1000× faster than microstructure-based models. Based on the sensitivity score, we found that the reduction percentage and the number of processing passes most significantly influence the final U-10Mo grain size and recrystallization percentage. Annealing time is important in controlling the refinement and coarsening of the final microstructure grains, while the influence of UC particles on U-10Mo microstructure properties is negligible. The proposed stratified stacking ensemble surrogate model can predict the U-10Mo grain size with a MAE that is 4 × smaller than obtained using the standard DNN method. With the flexibility of the stacking ensemble model, the framework developed in this study has good potential to generalize different alloy grain structure predictions under multi-pass thermomechanical processing.

## Methods

### Physics-based models

The physics-based model explicitly resolves the evolution of U-10Mo grain structures during multiple hot rolling and annealing passes. Specifically, FEM was developed for hot rolling, and a KMC Potts model was developed to simulate annealing^45,46^. The initial U-10Mo microstructures are generated synthetically using the open-source software Neper^47^. The generated tessellation statistics, such as initial grain size, standard deviation, and grain shape distributions, are fully customizable in Neper. This allows us to generate desired microstructures defined by the input parameters. The synthetic microstructure images then are imported into the FEM model for hot rolling. After strain analysis using FEM, the deformed foil microstructure results are passed to the Potts model for recrystallization simulation. In cases for which multiple hot rolling and annealing passes were simulated, the Potts simulation results were passed back to the FEM model for subsequent iterative rolling simulations.

The FEM model was applied to study the hot rolling process using the commercial Abaqus software. For the FEM simulation, we used two-dimensional plane strain compression to approximate the rolling process with straight boundaries in each representative volume element (RVE) domain. The U-10Mo grain microstructure RVE domain was explicitly imported and meshed using data generated by Neper. The UC particle data were generated for various cases by randomly distributing them across the whole RVE domain or along the grain boundaries. The mesh independence study indicated that the local strain levels in hot rolling simulations become independent with a mesh size smaller than 1 µm. To explicitly resolve the small UC particles and achieve an affordable cost to simulate the large RVE domain, a uniform mesh size of 1 µm was adopted for all the simulations in the FEM study.

The UC particles were considered isotropic in terms of material properties, with the Young’s modulus and Poisson’s ratio defined as 224.9 GPa and 0.288, respectively. The elastic behavior of the U-10Mo matrix was characterized by a 65 GPa Young’s modulus and 0.35 Poisson’s ratio. The polycrystalline grain structure and the anisotropy of the matrix were represented by randomly assigning an independent flow curve with a random ± 50% stress deviation from the baseline value^24^. After simulating a single rolling pass on the RVE, the results for each element are extracted for use in the next annealing step.

The equivalent plastic strain, phase, and grain orientation identification (ID) number generated by the FEM model are imported into the Potts model to simulate grain recrystallization and growth during the annealing step. The Potts model used in this study is a generalized version of the traditional Ising model with more than two states^13^. Evolution of the square grid of grain IDs, or “spins,” in the Potts is governed by the reorientation energetics among different grain IDs and the relative probability of these reorientation events. In principle, the total energy of a cell *E*_*i*_ can be described as follows:

1 $$E_{i} = U\left( {\varepsilon_{i} } \right) + \sum\limits_{j = 0}^{NN} {J_{ij} \left( {1 - \delta_{{s_{i} s_{j} }} } \right).}$$

$$U({\epsilon }_{i})$$ in the equation describes the energy associated with the deformation of cell *i* by an equivalent strain of $${\epsilon }_{i}$$. The $${J}_{ij}$$ term stands for the grain boundary energy, and the Kronecker delta $${\delta }_{{s}_{i}{s}_{j}}$$ describes whether the two neighboring cells $$(i,j)$$ have the same grain IDs ($${s}_{i},{s}_{j})$$. *NN* is a number of neighboring square lattice grid cells used to calculate deformation energy. The reorientation probability of a grain ID depends on the energy associated with the reorientation *ΔE* and the reorientation mobility *M*. The KMC simulation temperature *T*_*S*_ is a nonphysical term that controls grain boundary roughness in Potts model simulations^48,49^. The KMC algorithms weigh the likelihood of reorientation* p* according to the highest mobility *M*_*max*_ as follows:

2 $$p = \left\{ {\begin{array}{*{20}l} {\frac{{M_{ij} \left( T \right)}}{{M_{\max } }},} \hfill & {\Delta {\text{E}} \le {0}} \hfill \\ {\frac{{M_{ij} \left( T \right)}}{{M_{\max } }}e^{{\frac{ - \Delta E}{{T_{s} }}}} ,} \hfill & {\Delta E > 0} \hfill \\ \end{array} } \right.$$

The KMC implementations of the Potts model typically operate by selecting a reorientation randomly in the sequence of calculating reorientation probability, performing the reorientation, and advancing the clock^50^. In most cases, this “rejection-free” implementation of the Potts model achieves more efficient system evolution than “brute-force” implementations. Mapping the KMC algorithm time to actual physical time can be informed by comparing the simulation’s U-10Mo grain growth rate and recrystallization percentage to the empirically observed kinetics^13^. By doing so, the kinetics of both deformation-induced recrystallization and grain-boundary-curvature–driven grain growth can be accounted for in the simulation as a function of a given initial microstructure.

### Simulation parameters and conditions

In the hot rolling and annealing simulations, seven key parameters were investigated to identify their influence on the resulting U-10Mo foil microstructures. Those parameters can be divided into two groups. The first group is related to the processing conditions, such as single-pass hot rolling reduction percentage *R*, annealing time *T*, and the number of processing passes *N*. Hot rolling and annealing are carried out at a constant temperature of 700 °C in this work. The second group of parameters is related to the initial U-10Mo microstructure properties, including the initial average grain size *d*_*G*_ and its standard deviation *σ*_*G*_. The secondary-phase UC particle size *d*_*UC*_ and volume fraction *α*_*UC*_ are two other input parameters that potentially can affect U-10Mo foil properties.

**Figure 6:**
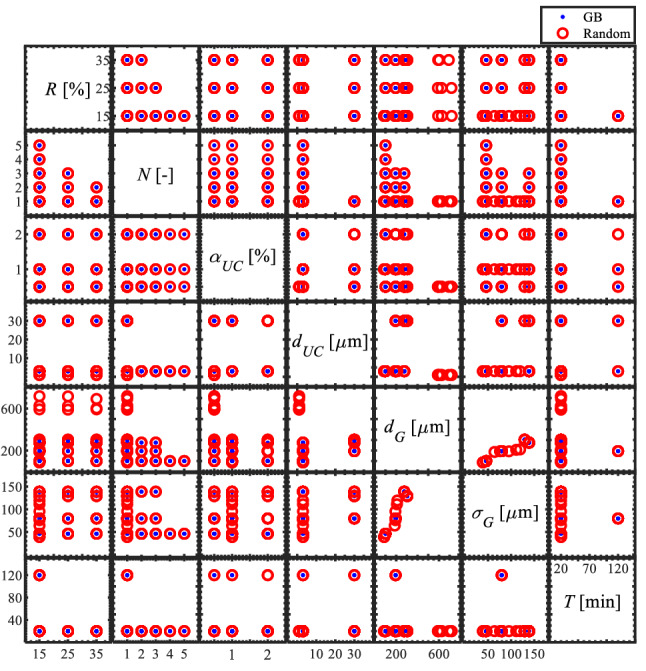
Distribution of the input parameters in the matrix plot form.

As shown in Fig. 6, distribution of the input parameter designs is visualized using a matrix plot. The blue dots represent the simulations with initial UC particles distributed on the grain boundaries, and the red circles stand for the UC particles distributed randomly across the RVE. The effect of rolling reduction is explored at three magnitudes: 15%, 25%, and 35%. The maximum number of hot rolling and annealing passes is five. The UC particle sizes are 3 μm and 30 μm, with a maximum volume fraction of 2%. Those parameters are selected according to practical ranges observed in the experiments. For the grain size, most cases have an average diameter of up to 300 μm, with a few cases having a size as large as 800 μm. The large grain size cases account for the abnormal grain growth during high-temperature homogenization^24^. For the annealing time *T*, the Potts model will run to either 20 or 120 min. Far fewer 120-min designs are analyzed than 20-min designs because of the higher computational costs for the longer runs.

After hot rolling and annealing, four parameters of interest are extracted from the simulated microstructures to investigate the physics and effects of the hot rolling and annealing processes. The final average grain ${d}_{G}^{F}$ and maximum grain size ${d}_{G,max}^{F}$ are calculated by collecting morphology information for all grains in the simulated RVE domain. Along with the grain size information, the recrystallization percentage *X* and JMAK exponent *n* also are calculated. Recrystallization during annealing forms a new set of strain-free grains with low dislocation density. Therefore, the recrystallization percentage *X* is closely related to the mechanical properties of the material. Along with annealing temperature and duration, the initial grain structure and UC impurities also can affect recrystallization. JMAK is used to describe transformation kinetics as a function of time *X*(*t*):

3 $$X\left( t \right) = 1 - \exp \left( { - kt^{n} } \right),$$

where *k* and *n* are time-independent constants that characterize grain nucleation and growth. The exponent *n* is selected as an output of interest because it characterizes time-dependent grain nucleation and growth that occurs during annealing^51^.

### Surrogate model and sensitivity analysis

To understand the influences of processing conditions and initial microstructures, surrogate models are developed for sensitivity analysis and predicting microstructures of the processed U-10Mo. The vector $\mathbf{x}=[{x}_{1},{x}_{2},\dots ,{x}_{n}]$ represents the input parameters (*n* = 7). The processed U-10Mo microstructure statistics can be predicted as ${\varvec{y}}={\varvec{f}}\left(\mathbf{x}\right)$, where ${\varvec{f}}$ represents the results predicted by the FEM and Potts model for hot rolling and annealing. The ***y*** in this case, is a four-dimensional vector that incorporates the relevant response output parameters listed in Fig. 1. The surrogate model denoted $\widehat{{\varvec{f}}}$ will be trained to approximate the high-fidelity simulation results ${\varvec{f}}(\mathbf{x})$ over the input domain *D* of interest. The model $\widehat{{\varvec{f}}}=[{\widehat{f}}_{1}\left(\mathbf{x}\right),{\widehat{f}}_{2}\left(\mathbf{x}\right),{\widehat{f}}_{3}\left(\mathbf{x}\right),{\widehat{f}}_{4}(\mathbf{x})]$ consists of four output components, and individual surrogate models will be trained correspondingly for each component. The MARS model will be used for the sensitivity analysis in the following discussions.

The MARS method is a popular nonlinear regression model used for data regression and sensitivity analysis^52,53^. Friedman first proposed the model in 1991 as a generalization of the recursive partitioning regression method^54,55^. The MARS surrogate model ${\widehat{f}}_{M}$ consists of a set of functions,

4 $$\hat{f}_{M} \left( {\mathbf{x}} \right) = \sum\limits_{i = 1}^{M} {c_{i} B_{i} \left( {\mathbf{x}} \right)} ,$$

where the basis function ${B}_{i}(x)$ can be a constant, a hinge function, or a product of two or more hinge functions to account for the input parameter interactions. Users can specify the order of interactions by selecting the maximum number of hinge functions for a product. The ${c}_{i}$ are coefficients for the corresponding basis function ${B}_{i}\left(x\right)$, which can be determined by minimizing the residual sum of squares (RSS). The RSS is defined as:

5 $$RSS={\sum }_{i=1}^{n}{\left({y}_{i}-\hat{f}_{M}\left({\mathbf{x}} \right)\right)}^{2}.$$

The selection of the basis functions is divided into two steps: (1) forward selection and (2) backward deletion. The basis functions are selected and added sequentially according to which function can reduce the training error the most in the forward selection. This step will continue until the function number reaches its pre-specified maximum. Then, backward deletion is done to mitigate the overfitting issues in the forward selection. In this step, the generalized cross-validation (GCV) score^55^ is used as the backward deletion criteria, which is calculated as:

6 $$GCV = \frac{1}{N}\sum\limits_{i = 1}^{N} {\left[ {y_{i} - \hat{f}_{M} \left( {\mathbf{x}} \right)} \right]} /\left( {1 - \frac{C\left( M \right)}{N}} \right)^{2} ,$$

The term in the numerator is the mean RSS of the MARS model, and in this case, *N* stands for the total number of simulations. *C*(*M*) is defined as the effective number of parameters, which penalizes the addition of basis functions^56^. M is the non-constant basis function number.

To quantify the influence of each input parameter on the final microstructure properties, the sensitivity score is calculated based on the GCV scores. For each input parameter *x*_*i*_, a separate score GCV(*M*\*x*_*i*_) will be calculated by removing the basis functions in the trained model ${\widehat{f}}_{M}(\mathbf{x})$, which involves the *x*_*i*_. By subtracting the full-model GCV, the sensitivity score for each input parameter ${x}_{i}$ can be calculated as:

7 $$s_{i} = GCV(M\backslash x_{i} ) - GCV(M).$$

A larger score *s*_*i*_ means the model error becomes larger when the corresponding input parameter is removed. This indicates that the selected input parameter would significantly influence the output results.

The stacking ensemble method was proposed by Wolpert in the early 1990s with the strategy of combining multiple sub-level surrogate models to reduce the model biases and to improve the prediction accuracy^43^. The stacking ensemble method generally consists of first-level submodels and a second-level meta-model^57–59^. In the algorithm, multiple first-level submodels will be trained on the whole dataset separately. The second-level metamodel will then combine the first-level model predictions to give a final model prediction. Compared to the weighted-average ensemble method, the stacking ensemble can determine the weight of each first-level model prediction using more advanced algorithms in the second-level model. This could potentially improve the whole model accuracy. For the first-level prediction, the submodels can be the same or mixed types of surrogate model. In this study, the DNN was selected as the base model because it is flexible and can scale with the size of the dataset. Six first-level submodels are trained for the stacking ensemble.

Considering the complex physics involved in hot rolling and annealing, a more robust stratified stacking ensemble method is proposed to improve grain structure prediction accuracy. The structure of the proposed stratified stack ensemble model can be found at the Fig. 1. The idea of this method is to split the training dataset into two groups $\{{\mathbf{x}}^{A},{\mathbf{x}}^{B}\}$ according to the output value $y=f(\mathbf{x})$ quantity:

8 $$\left\{ \begin{gathered} {\mathbf{x}}^{A} \subset {\mathbf{x}} \mid f\left( {{\mathbf{x}}^{A} } \right) < \frac{{f\left( {\mathbf{x}} \right)_{\max } + f\left( {\mathbf{x}} \right)_{\min } }}{2}, \hfill \\ {\mathbf{x}}^{B} \subset {\mathbf{x}} \mid f\left( {{\mathbf{x}}^{B} } \right) > \frac{{f\left( {\mathbf{x}} \right)_{\max } + f\left( {\mathbf{x}} \right)_{\min } }}{2}. \hfill \\ \end{gathered} \right.$$

Each split training data is used to train half of the first-level submodels separately. Predictions of the submodels will then be combined in the second-level metamodel to make the final predictions. In the prediction step, test data will be stratified by comparing the minimum distances to the two training datasets $\{{{\varvec{x}}}^{A},{{\varvec{x}}}^{B}\}$ and then fed into the corresponding first-level models. Recrystallization can happen quickly and cause either a small or large fraction of grains to be recrystallized at a given annealing time. With the stratified stacking ensemble method, performance can be enhanced by training each group of first-level models specifically for the divided training parameter space.

## Data Availability

The raw/processed data required to reproduce these findings cannot be shared at this time as the data also forms part of an ongoing study.
